# The Role of Personality Traits and Decision-Making Styles in Career Decision-Making Difficulties

**DOI:** 10.3390/bs15020159

**Published:** 2025-02-01

**Authors:** Mine Aydemir Dev, Nuran Bayram Arlı

**Affiliations:** Department of Econometrics, Bursa Uludag University, Bursa 16059, Türkiye; nuranb@uludag.edu.tr

**Keywords:** career decision-making difficulties, decision-making styles, personality traits

## Abstract

Career decisions are one of the most important decisions individuals make in their lives. These decisions are multifaceted and complex, making them a challenging process, particularly for students. This creates obstacles for students navigating the career choice process. The aim of this study is to examine career decision-making difficulties within the framework of personality traits and decision-making styles. The target group of this study is undergraduate students studying at a public university. For this research, we collected data through online questionnaire forms. The questionnaire comprised demographic questions and three scales: career decision-making difficulties, personality traits, and decision-making styles. A total of 505 students (63.2% female) participated in the questionnaire. The research model was tested using path analysis. We also conducted network analysis in order to better understand and visualize the relationships between the sub-dimensions of the scales. The findings showed significant direct relationships between career decision-making difficulties and personality traits (conscientiousness, openness, neuroticism, and agreeableness) and decision-making styles (rational, dependent, avoidant, and spontaneous). According to the path analysis result, neurotic personality traits were positively correlated with career decision-making difficulties, while openness to experience, agreeableness, and conscientiousness were negatively correlated with them. The relationship between dependent, avoidant, and spontaneous decision-making styles and career decision-making difficulties was positive. These results provide valuable insights into the factors that shape career decisions, helping students make more informed choices and manage their career paths effectively.

## 1. Introduction

Career decisions are one of the most important decisions that individuals make. Proper and good career choices contribute to the effective use of resources and to increased satisfaction and productivity in working life by matching individuals with occupations that match their skills and interests. However, career choice is known to be a multidimensional and complex process and is often a challenging decision-making process, especially for students. Even after completing their education, there are many students who have not been able to set a career goal or develop a plan for their life. Students’ career choices are an important issue not only for students but also for families and educators.

Career decision-making involves different experiences for each individual. While some individuals may find this process an exciting and fulfilling opportunity, others may face various challenges. While some individuals are driven by an innate orientation and interest in their career choices, others may experience uncertainty and indecision in the decision-making process. This diversity shows that the process of making career decisions is not only about challenges but also offers opportunities for individuals to discover their potential and realize themselves. However, in today’s contemporary labor market, choosing a career path has become increasingly difficult for adolescents and young adults, and many difficulties are encountered in the career decision-making process (Bimrose & Mulvey, 2015). Career indecision is a concept used to describe the difficulties and uncertainties that individuals face when making career decisions (Gati et al., 1996). This issue, also called *career decision-making difficulties* (Gati et al., 1996), refers to individuals’ inability to make a career-related decision or having difficulties in this process. This decision may involve choosing a profession and related education, then choosing a job and then whether to stay in a job or not.

Decision-makers (students) need to better understand the challenges and reasons behind career decisions in order to make more informed career decisions. Faced with such critical decisions, students often face a variety of challenges that make these decisions difficult or lead to the selection of sub-optimal alternatives. Therefore, it is of great importance to use different tools and guidance methods to help students make decisions that best suit their needs, abilities, and personalities (Kulcsár et al., 2020). It is known that career decisions are not limited to rational considerations and are influenced by individual factors such as personality traits, decision-making styles, values, interests, and abilities but also by non-individual factors (family, society and environment, etc.). This study examines career decision-making difficulties within the framework of *personality traits* and *decision-making styles*.

Following this perspective, it is suggested that decision-making styles may play a mediating role in the relationship between personality traits and career decision-making difficulties. Personality traits shape the way they gather and evaluate information and make decisions, leading to their preference for certain decision-making styles (Eser, 2022). These different decision-making styles can be effective in determining the type and severity of difficulties that individuals face in career decision-making.

Decision-making styles provide an important intermediate mechanism in explaining how tendencies arising from personality traits are manifested in the career decision-making process. In this context, since the effect of personality traits on career choice difficulties is an indirect process rather than a direct effect, decision-making styles act as a mediator. While testing this mediating role, the current study aims to explain how personality traits affect career decision-making difficulties. Therefore, decision-making styles provide an important framework for understanding how dispositions stemming from personality traits are reflected in the difficulties in individuals’ career decision-making processes. By testing this mediating role, our research aims to provide a better understanding of individuals’ career decision difficulties.

Studies on career decision-making, personality traits, and decision-making styles are present in the literature. However, studies examining the relation of personality traits and decision-making styles to career decision-making difficulties are limited. To our knowledge, there is no study in the literature that examines all three together. In addition, the mediating role of decision-making styles in the relationship between personality and career difficulties has never been examined. This suggests that there is a need for further research on career decision-making difficulties. This study highlights this gap in the field and aims to obtain results with an empirical application and fill this gap. Based on this, the three research questions to be answered are as follows:

- Which personality traits are associated with career decision-making difficulties?
- Which personality traits and decision-making styles are related?
- What role do decision-making styles play in the relationship between personality traits and career decision-making difficulties?

University students face several challenges in making career decisions, and this decision-making process is known to be a developmentally complex task. The impact of poor career choices can extend to the student’s work environment and relationships with people in their social environment, making this process stressful. Addressing these challenges is an important starting point for career planning. At the same time, today’s rapidly changing business world forces students to consider career paths that are multifaceted and full of uncertainties (Parola & Marcionetti, 2024). Accordingly, understanding the factors that influence students’ career indecision is thought to help them manage their career journeys more conscientiously and effectively. In particular, individual personality traits and decision-making styles play an important role in understanding career decisions. The purpose of this study is to examine the relationship between these two concepts and career decision-making difficulties. In other words, the main purpose of this study is to examine the effects of personality traits and decision-making styles on career decision-making difficulties.

## 2. Theoretical Framework and Research Hypothesis

Gati et al. (1996) presented a theoretical framework for understanding the challenges individuals face when making career decisions and developed a taxonomy of career decision-making challenges based on decision theory. This framework aims to measure the specific challenges that individuals face when making career decisions. This taxonomy classifies the difficulties faced by individuals when making career decisions under three main categories: a *lack of readiness*, a *lack of information,* and *inconsistent information*.

The first category, a lack of readiness, includes a lack of motivation, general indecision, and dysfunctional beliefs about the process. The second category, a lack of information, is explained by the lack of sufficient information about oneself and the profession. This category includes a lack of information about the process, a lack of information about oneself, a lack of information about occupations, and a lack of information about how to obtain additional information. The last category, inconsistent information, is related to unreliable information. This category includes unreliable information, internal conflicts, and external conflicts (Gati et al., 1996). These three main categories can be further subdivided into more detailed subcategories to help identify the specific challenges that individuals face in their vocational decision-making processes.

Based on this taxonomy, Gati et al. (1996) developed the Career Decision-Making Difficulties Questionnaire (CDDQ). The CDDQ aims to systematically assess the problems individuals experience in making decisions and to identify the source of these problems. Particularly in career counseling, it allows for the development of strategies that are appropriate to the individual’s difficulties. The validity and reliability of the CDDQ have been tested in many cultures and studies (Lancaster et al., 1999; Gati et al., 2000; Arnold, 2003; Tien, 2005; Amir et al., 2008; Vahedi et al., 2012; Sovet et al., 2015; Babarović & Šverko, 2019; Levin et al., 2023), and it is an important tool widely used by career counselors. This tool provides a comprehensive assessment to identify the source of difficulties and helps to guide individuals more effectively in career counseling (Gati et al., 1996; Xu & Bhang, 2019).

### 2.1. Career and Personality Traits

Personality traits are conceptualized as a set of stable individual differences in people’s motivational responses to restricted classes of environmental stimuli. There are different models of personality traits in the field of psychology (Bayram & Aydemir, 2017). Generally, the Five-Factor Theory (J. Costa & Paul, 1996; P. T. Costa & McCrae, 1999; Whiteside & Lynam, 2001; Boyle, 2008; McCrae & Costa, 2008; Soto & Jackson, 2013; Novikova & Vorobyeva, 2019) is one of the most widely used theories in explaining personality traits. This theory has an integrative function as it can represent various personality description systems in a common framework. This theory, which provides an appropriate framework for examining the effects of personality traits on decision-making, deals with personality in five main dimensions: *extraversion, conscientiousness, agreeableness, neuroticism (emotional instability),* and *openness to experience*.

Extraversion is indicated by a higher degree of sociability, assertiveness, and talkativeness. Conscientiousness shows being disciplined, organized, and achievement-oriented. Agreeableness indicates being helpful, cooperative, and sympathetic towards others. Neuroticism, which is a core dimension of many personality models, is defined as a predisposition toward negative affective states such as depression, anxiety, anger, and sham (Miller & Pilkonis, 2006). Openness reflects a strong intellectual curiosity and a preference for novelty and diversity (Komarraju & Karau, 2005). Many different concepts have been discussed together with personality traits. One of them is career indecision or, in other words, career decision-making difficulties.

*Extraversion*, one of the personality traits, was found to be associated with career indecision (Di Fabio et al., 2012, 2015). Extraverted individuals were found to be more successful in decision-making difficulties such as inconsistent information (Di Fabio & Palazzeschi, 2012). Extraverted individuals’ communication skills and social connections make it easier for them to learn more about career options. This is associated with reduced uncertainty in the career decision-making process and improved ability to overcome decision-making difficulties. It has been observed that especially extraverted participants experience less career indecision (Di Fabio et al., 2015; Martincin & Stead, 2015; Akbar et al., 2023). According to studies, it is known that individuals with a high sense of *conscientiousness* make more careful and conscious decisions and reduce the difficulties in career decision-making (Di Fabio & Palazzeschi, 2012). Conscientious individuals evaluate alternatives more carefully and make more informed choices through disciplined and structured decision-making processes. This characteristic is associated with reduced complexity in career decision-making. Studies have shown that personality traits such as extraversion and conscientiousness reduce career indecision (Öztemel, 2014; Martincin & Stead, 2015).

*Neuroticism* is known to have a strong positive relationship with career indecision (Burns et al., 2013; Martincin & Stead, 2015; Di Fabio et al., 2015). Neurotic personality traits are negatively and significantly associated with the lack of readiness and inconsistent information dimensions of career indecision (Di Fabio et al., 2012). Neurotic individuals have more difficulty in decision-making processes because they experience higher stress in the face of uncertainties and unfavorable situations. This increases decision-making difficulties, especially a lack of information and inconsistent information. It is known that individuals with high emotional balance have less difficulty in decision-making processes, especially in the dimensions of a lack of readiness and a lack of information. In other words, high neuroticism levels are associated with more career indecision (Öztemel, 2014; Akbar et al., 2023).

Previous studies have concluded that the *openness to experience* and *agreeableness* personality traits have a negative relationship with career indecision (Lounsbury et al., 1999; Di Fabio & Palazzeschi, 2009, 2012; Al-Kalbani et al., 2011; Smith, 2011; Di Fabio & Saklofske, 2014; Di Fabio et al., 2015; Martincin & Stead, 2015). Individuals who are open to experience can evaluate their options from a broader perspective and make informed decisions thanks to their innovation and analytical thinking skills. This characteristic can be effective in reducing career decision-making difficulties. The negative relationship between the openness to experience and agreeableness personality traits and career indecision indicates that these individuals are able to set clearer career goals and make effective decisions. Adaptive individuals facilitate decision-making and set clearer goals thanks to their ability to cooperate and their tendency to seek support from others. While openness to experience provides openness to innovation and analytical skills, agreeableness supports it with cooperation and consensus-building skills. These traits allow individuals to better define their professional interests and abilities and make career decisions more easily. Therefore, individuals with these personality traits are expected to have stronger self-awareness and decision-making skills that reduce career indecision.

In general, through a review of studies on personality traits and career decision-making difficulties, it is revealed that individuals with neurotic personality traits experience more career indecision, those with extraverted personality traits experience less career indecision, and the personality traits of conscientiousness, openness, and agreeableness are negatively related to career indecision. The findings obtained from these studies indicate that personality traits play an important role in individuals’ career decision-making processes and should be taken into consideration in career counseling. These findings show the effects of each personality trait on decision-making processes and the importance of considering these effects in career counseling.

### 2.2. Career and Decision-Making Styles

Decision-making plays an important role in people’s lives. Decision-making is generally defined as the characteristic mode of individuals in perceiving and responding to decision-making tasks. A decision-making style is defined as a situation that includes the approach, reaction, and action of an individual who is about to decide (Leonard et al., 1999; Dewberry et al., 2013; Thunholm, 2004; Bayram & Aydemir, 2017). Scott and Bruce’s (1995) General Decision-Making Styles (GDMSs) model explains how individuals structure their decision-making processes. The model includes five different decision-making styles: *rational*, *intuitive*, *dependent*, *avoidant*, and *spontaneous* decision-making styles.

The *rational* decision-making style involves making logical, analytical, and planned decisions. The *intuitive* style involves making decisions instinctively and quickly. The *dependent* style involves making decisions based on others. The *avoidant* style refers to avoiding decisions and postponing decision-making processes. The *spontaneous* style shows quick and often unplanned decision-making. According to Scott and Bruce, individuals usually have all five styles at different levels, but one style is usually dominant. Career decisions are considered as a special category of decision-making and have long-term effects on individuals’ lives and usually involve high levels of uncertainty, anxiety, and stress. Such decisions differ from other types of decisions in that they have a direct impact on an individual’s identity formation, life satisfaction, and social status. Career decisions can affect the opportunities, lifestyle, and financial status of the individual for years to come. Career decisions are often shaped by an individual’s dominant decision-making style. Whether a particular decision-making style is more critical than others in career decision-making processes may vary depending on the context, personal characteristics, and complexity of the decision. Although there are studies examining decision-making styles and different concepts, there are a limited number of studies on career indecision.

Chong and Tan (2019) examined the relationship between decision-making styles and career indecision in detail. The study was conducted with a sample of senior university students in Malaysia and found that except for the rational style, the other four decision-making styles (dependent, intuitive, avoidant, and spontaneous) were significantly positively related to career indecision. In particular, the dependent and avoidant decision-making styles were found to have the strongest positive effects on career indecision. In contrast, the rational decision-making style was not found to have a significant relationship with career indecision. These findings make an important contribution to the literature by revealing the effect of different strategies used by individuals in decision-making processes on career ambivalence. Additionally, in studies where the relationships between career and decision-making styles are sought, scales such as the ADMQ (Adolescent Decision-Making Questionnaire) and MDMQ (Melbourne Decision-Making Questionnaire) were mostly used instead of GDMSs (Mann et al., 1997; Tuinstra et al., 2000; Di Fabio & Palazzeschi, 2012; Pečjak et al., 2019).

### 2.3. Personality Traits and GDMSs

Studies in the literature show that there are significant relationships between personality traits and decision-making styles. Extraversion, openness, agreeableness, and conscientiousness were found to be positively related to the rational and intuitive decision-making styles, whereas openness showed a negative relationship with the dependent decision-making style (Narooi & Karazee, 2015; Bayram & Aydemir, 2017). In another study, it was found that extraversion positively affected the intuitive and spontaneous decision-making styles, openness to experience positively affected the intuitive decision-making style, agreeableness positively affected the dependent decision-making style, and conscientiousness positively affected the rational decision-making style (Riaz et al., 2012). It was concluded that neurotic personality traits and the avoidant decision-making style were positively related (Riaz et al., 2012; Narooi & Karazee, 2015).

In another study that examined personality traits and decision-making styles, it was found that extraverts tended to have a lower rational decision-making style, higher agreeableness and conscientiousness were significantly associated with the rational decision-making style, more extraversion and openness to experience were significantly associated with a higher intuitive decision-making style, and higher agreeableness and conscientiousness were significantly associated with a lower intuitive style. In the related study, none of the personality traits were found to be significantly associated with the avoidant decision-making style (El Othman et al., 2020). Another study found that a neurotic disposition was a strong predictor of perceived problem-solving ability, a dependent decision-making style, and both affective and informational antecedents of career indecision. In addition, relationships were found between personality traits and career decision-making styles. It was found that the conscientiousness personality type was positively related to rational decision-making and negatively related to intuitive decision-making, while the openness to experience personality trait was negatively related to dependent decision-making (Chartrand et al., 1993). A general summary of the relations between decision-making and personality traits is presented in Table 1.

### 2.4. Research Model

**Figure 1:**
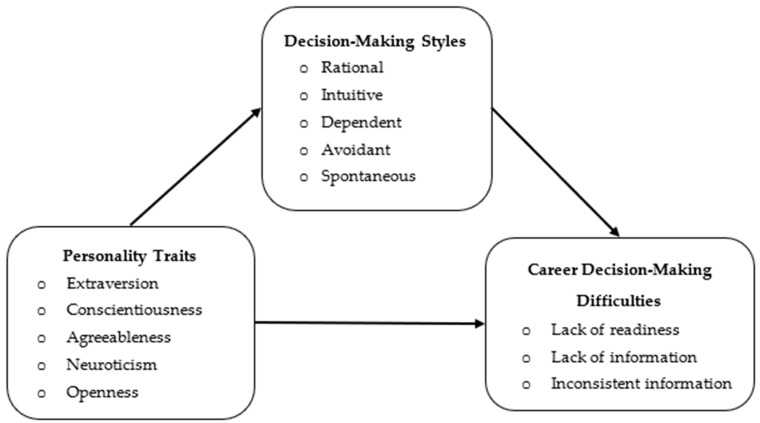
Research model.

The main focus of this research is to explore the role of personality traits and decision-making styles in career decision-making difficulties. Accordingly, the conceptual research model analyzed in this study is presented in Figure 1.

There are four hypotheses to be tested within the scope of this research:

**H1.**  *There are significant relationships between personality traits and decision-making styles*.

**H2.**  *There are significant relationships between personality traits and career decision-making difficulties*.

**H3.**  *There are significant relationships between decision-making styles and career decision-making difficulties*.

**H4.**  *Decision-making styles play a mediating role in the relationship between personality traits and career decision-making difficulties*.

The effects of each personality type and decision-making style’s sub-dimensions are examined in detail within the scope of the model.

## 3. Materials and Method

### 3.1. Participants

The target group of this study is undergraduate students studying at a state university in Türkiye. A convenience sampling method was used to collect the data with a questionnaire form containing scales and demographic information. The questionnaires were conducted online via Google forms and filled out individually by volunteer participants. The questionnaire form for this study was approved by Bursa Uludag University Social and Human Sciences Research and Publication Ethics Committee (2024-09).

The number of students actively studying at the undergraduate level at the university in 2024 was about 40,000. When conducting this study, it was sufficient to reach 384 students at the 95% confidence level (Bayram, 2016). It is known that there is a direct relationship between the sample size and the reliability of the estimation. Therefore, this study aimed to exceed this number. The final sample was 505 students. This sample size is sufficient to represent the main population.

### 3.2. Measurement Tools

In this study, a questionnaire form was used for data collection. The questionnaire included scales measuring career decision-making difficulties, decision-making styles, personality traits, and questions measuring demographic characteristics. Validity and reliability studies of the scales in Turkish had been previously conducted and were applied accordingly. Participation in the questionnaire was carried out on a voluntary basis, and no participation fee was provided. The questionnaire was completed in approximately 15 min. Information about the scales is presented below.

#### 3.2.1. Big Five Personality Traits

The Five-Factor Inventory was developed by John et al. (1991). Turkish validity and reliability studies were conducted by Sümer and Sümer (2005) and Alkan (2007). It is quite short for a multidimensional personality inventory. The five personality traits measured are extraversion, agreeableness, conscientiousness, neuroticism, and openness to experience. There are 44 items in the scale. The items are scored on a 5-point Likert scale ranging from strongly disagree (1) to strongly agree (5). The following are some sample items from the inventory: “*I am talkative*” and “*I am open to new, original ideas*”. High scores indicate that the respective personality dimension is high. Sümer et al. (2005) found that the reliability coefficients of the five-factor personality dimensions ranged between 0.64 and 0.77.

#### 3.2.2. General Decision-Making Styles (GDMSs)

This scale was developed by Scott and Bruce (1995). It measures five different decision-making styles: rational, intuitive, dependent, avoidant, and spontaneous. The validity study of the Turkish version of the scale was conducted by Taşdelen (2002). This scale consists of 24 items. All items are scored on a 5-point Likert scale ranging from strongly disagree (1) to strongly agree (5). The following are some sample items from the scale: “*I make decisions in a logical and systematic way*” and “*My decision making requires careful thought.*” Higher scores indicate a higher level of the respective decision-making style. In the study of Scott and Bruce (1995), reliability coefficients were calculated in different samples and were found to be between 0.77 and 0.94. Taşdelen (2002) calculated the Cronbach’s alpha coefficients for the reliability of the scale as 0.76 for rational, 0.78 for intuitive, 0.76 for dependent, 0.79 for avoidant, and 0.79 for spontaneous.

#### 3.2.3. Career Decision-Making Difficulties Questionnaire (CDDQ)

The scale, originally developed by Gati et al. (1996) and Gati and Saka (2001a, 2001b), classifies career decision-making difficulties into three main categories and ten subcategories: lack of readiness (lack of motivation, general indecision, dysfunctional beliefs), lack of information (lack of information about the process, lack of information about the individual, lack of information about occupations, and lack of information about how to obtain additional information), and inconsistent information (unreliable information, internal conflicts, and external conflicts). The scale consists of 34 items. The original items are rated on a 9-point scale from 1 (“Does not describe me well”) to 9 (“Describes me well”). Turkish validity studies were conducted by Bacanlı (2016) by adapting it to high school students. The scale adapted to Turkish is evaluated with a 5-point Likert scale. The following are some sample items from the scale: “*I know that I have to choose a career, but I don’t have the motivation to make the decision now*” and “*It is usually difficult for me to make decisions*” (Levin et al., 2023). The higher the score obtained from the scale, the higher the level of career decision-making difficulty.

Gati and Saka (2001a) calculated the Cronbach’s alpha internal consistency coefficients for the reliability of the original scale as 0.62 for lack of readiness, 0.88 for lack of information, 0.87 for inconsistent information, and 0.91 for the total scale. This study was conducted with 1843 Israeli adolescents. Another study conducted by Bacanlı (2016) with 2509 Turkish adolescents calculated the Cronbach’s alpha internal consistency coefficients for the Turkish reliability as 0.45 for lack of readiness, 0.90 for lack of information, 0.84 for inconsistent information, and 0.90 for the total scale.

### 3.3. Analysis

Descriptive statistics and Cronbach’s alpha values for the reliability of the scales are reported. Path analysis was performed to estimate the research model, and *X*^2^*/df*, *RMSEA*, *SRMR*, *GFI*, and *CFI* indices are reported to examine the fit of the estimated model. Network analysis was used to better understand and visualize the relationships between variables. IBM SPSS 28.0 was used for descriptive statistics, correlations, and Cronbach’s alpha values, AMOS 23.0 was used for path analysis to test the hypotheses, and JASP 0.18.3.0 was used for network analysis.

#### 3.3.1. Path Analysis

Path analysis is a statistical method that uses path diagrams as an extension of the regression model to test causal relationships between variables and to visualize these relationships. Researchers perform regression analysis for each dependent variable in the model, compare the results with the observed correlation matrix, and evaluate the goodness of fit of the model. The ability to examine direct and indirect effects and to analyze multiple dependent and independent variables simultaneously is among the most powerful aspects of path analysis. Path analysis is nowadays generally performed with Structural Equation Modeling (SEM), and unlike SEM, it focuses on observed variables (Stage et al., 2004; Garson, 2013).

#### 3.3.2. Network Analysis

The scales included in this research have more than one sub-dimension. Because there are complex relationships among these numerous variables, network analysis was used to better understand and visualize these relationships. Network analysis provides deeper insight into complex relationships and connections. In addition, the centrality measures used in network analysis help to determine the importance of certain variables or nodes in the network (Zhai et al., 2024). The network structure was calculated using 1000 bootstrapping methods with correlation estimation at a 95% confidence interval.

In the network approach, each feature is treated as a node that can connect to other nodes and interact through these connections (edges). All of these connections between nodes make up the entire network (Trahair et al., 2020). Betweenness centrality emphasizes that a node acts as a bridge between other nodes in the network and is an important waypoint. Closeness centrality indicates a node’s proximity to other nodes in the network, thus its capacity to interact faster over short paths. Strength centrality shows the connections, strong relationships, importance, and influence of a node with other variables in the network. A higher expected influence suggests that a node plays a more significant role as a risk factor when the scores are positive or as a protective factor when the scores are negative. Each metric plays a critical role in understanding the nodes’ strategic positions and interactions in the network (Ochnik et al., 2024).

## 4. Findings

### 4.1. Preliminary Analyses

This section presents the preliminary analysis of the measurement tools used in this study. Table 2 shows the number of items, means, and standard deviations of each scale. In addition, the reliability coefficients (Cronbach’s alpha) of each scale were calculated to assess the consistency of the measurements. Reliability coefficients were used to determine the internal consistency of the scales, and the values obtained showed that the scales had sufficient reliability.

While Gati and Saka (2001a) evaluated the reliability of the original CDDQ, Bacanlı (2016) calculated Cronbach’s alpha internal consistency coefficients for different dimensions to test the Turkish reliability of the scale. The results obtained for this study were similar to those of Gati and Saka (2001a) and Bacanlı (2016), and the lack of readiness dimension had a low Cronbach’s alpha value, while the other dimensions had high values.

The correlation values between the scales were calculated to examine the relationships between the variables. Table 3 presents the correlation values. Moderately significant correlations were found between personality traits and most of the decision-making styles. Likewise, moderately significant correlations were found between personality traits and most of the career decision-making difficulties. When the relationships between decision-making styles and career decision-making difficulties are examined, it is seen that the intuitive decision-making style has no significant correlation with any career decision-making difficulty, but the other styles have moderately significant relationships.

### 4.2. Descriptive Statistics

This section presents descriptive statistical analyses of the research data. Descriptive statistics are used to understand the general characteristics of the data set and to provide a basic overview of the participants’ responses. In order to better understand the overall structure of the data, descriptive summaries such as frequency distributions and percentiles are presented in Table 4.

Of the 505 participants, 63.2% were female and 36.8% were male. When the distribution of the participants according to their grade levels was analyzed, 22.8% were first-grade students, 22.2% were second-grade students, 24.8% were third-grade students, and 30.3% were fourth-grade students. The rate of those who had difficulty in making career decisions was 62.4%, and 37.6% stated that they did not have any difficulty in making decisions. While 73.1% of the participants stated that they chose their field of study conscientiously, 26.9% stated that they did not make this choice conscientiously.

In this study, which was conducted with students from a state university, participants were from all grades, with more than half of the participants being women. A large number of them were uncertain about making a career decision. A significant number of participants have conscientiously chosen their field of study.

### 4.3. Network Analysis Results

In this study, network analysis was conducted in order to better understand and visualize the relationships between the sub-dimensions of personality traits, decision-making styles, and career decision-making difficulties. The relationships between the dimensions are complex, and it is important to see the interactions between these dimensions. Network analysis clearly shows the connections between these sub-dimensions, revealing which characteristics have a strong relationship with each other or function more independently. It also provides a visual representation of how the dimensions influence each other.

**Figure 2:**
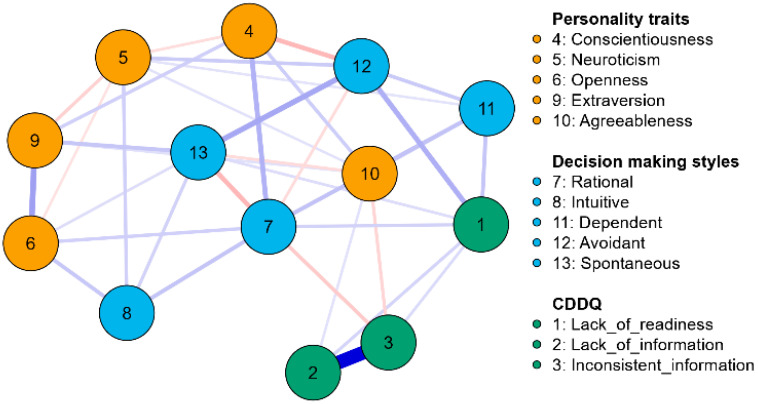
Network graph.

The network graph is shown in Figure 2. The red lines in Figure 2 represent negative correlations, and the blue lines represent positive correlations. Moreover, the thickness of the lines indicates the strength of the correlations.

The results of the network analysis shown in Figure 2 indicate that there are significant relationships between personality traits, GDMSs, and CDDQ dimensions. The relationships between these three types of variables suggest that personality traits have a direct relationship with individuals’ decision-making styles, and personality traits are indirectly related to decision-making difficulties. In particular, the strongest connection was found between 2 (lack of information) and 3 (inconsistent information) in the CDDQ. Moreover, the strongest connections between the sub-dimensions of the scales other than their own were found between 1 (lack of readiness) and 12 (avoidant) and between 9 (extraversion) and 13 (spontaneous).

**Figure 3:**
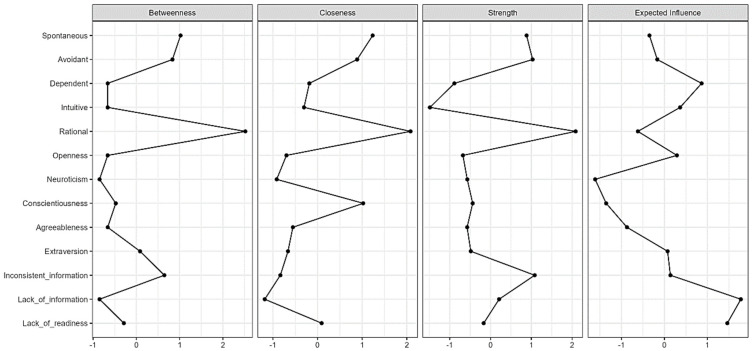
Centrality plot.

Centrality analysis was performed to evaluate the centrality of nodes in the integrated network and is presented in Figure 3. Accordingly, a lack of information, a sub-dimension of the CDDQ, was found to have the highest Expected Impact (EI) index. In addition, the rational style of GDMSs displayed the highest scores in strength, closeness, and betweenness centrality. In other words, the rational decision-making style emerged as the most central variable in all centrality indices, followed by the spontaneous style. This shows that the rational style plays a critical role in connecting other nodes or acts as a bridge and is close to other nodes in all dimensions. GDMSs that do not play a critical role appear to have a limited impact on the overall information flow and connectivity of the network due to their low centrality values.

### 4.4. Path Analysis of the Research Model

Path analysis was conducted to examine the relationships between the main variables of this study in depth. Path analysis is a structural modeling method used to determine the direct and indirect effects between variables. Based on the research model, this analysis provides a clearer picture of the relationships between variables. In addition, the results of the path analysis and the validity of the model are discussed together with the fit indices. Only statistically significant results are reported to provide a clear and focused presentation of the findings. The estimated model results are shown in Table 5.

The goodness-of-fit values calculated for the estimated path analysis were *X*^2^/*df* = 3.574; *GFI* = 0.97; *CFI* = 0.96; *RMSEA* = 0.071; and *SRMR* = 0.051. The obtained goodness-of-fit values are at an acceptable level. Table 5 reveals the existence of significant relationships between personality traits and decision-making styles. Extraversion showed a negative relationship with the rational (*β* = −0.091, *p* = 0.034) and avoidant (*β* = −0.174, *p* = 0.007) decision-making styles, while it showed a positive relationship with spontaneous decision-making (*β* = 0.225, *p* < 0.000). In the literature, it has been reported that extraverted individuals think less analytically but prefer to make faster and more spontaneous decisions. This tendency may be related to the fact that extraverts exhibit a more general, flexible, and “less concrete” style in both cognitive and verbal communication (Beukeboom et al., 2013). However, this may also reflect the fact that they minimize their tendency to plan for the future, which is another aspect of their rational decision-making process. Moreover, it can be considered as a noteworthy positive characteristic that extraverted individuals do not avoid making decisions and, on the contrary, play an active role in rapid decision-making processes. Agreeableness positively contributes to rational (*β* = 0.109, *p* = 0.037) and dependent (*β* = 0.278, *p* < 0.000) decision-making styles, while it is negatively related to avoidant (*β* = −0.160, *p* = 0.041) and spontaneous (*β* = −0.186, *p* = 0.005) decision-making tendencies. It can be said that individuals with high levels of agreeableness adopt a decision-making approach that is more planned and open to seeking support.

Conscientiousness is strongly positively associated with rational decision-making (*β* = 0.409, *p* < 0.000) and negatively associated with the spontaneous (*β* = −0.428, *p* < 0.000) and dependent (*β* = −0.166, *p* = 0.011) decision-making styles. This suggests that conscientious individuals act more analytically and deliberately but avoid dependent or spontaneous reactions. Moreover, conscientiousness showed a positive relationship with intuitive decision-making (*β* = 0.121, *p* = 0.037), indicating that these individuals are able to balance both analytical and intuitive approaches. Neuroticism was positively associated with the dependent (*β* = 0.147, *p* = 0.003) and intuitive (*β* = 0.180, *p* < 0.000) decision-making styles. Neuroticism is associated with mood swings and inefficient emotional regulation skills, which can influence decision-making in certain problem-solving situations (Kokkonen & Pulkkinen, 2001). These emotional fluctuations may lead neurotic individuals to rely on external advice or be more susceptible to others’ suggestions, particularly in high-stakes contexts such as career decision-making.

Openness to experience was positively associated with rational (*β* = 0.217, *p* < 0.000) and intuitive (*β* = 0.345, *p* < 0.000) decision-making and negatively associated with an avoidant decision-making tendency (*β* = −0.280, *p* < 0.000). This suggests that individuals with openness to experience tend to think more innovatively, analytically, and intuitively and move away from avoidance behavior. In general, these results reveal the different effects of personality traits on decision-making styles and show that it is possible to make sense of individuals’ preferences in decision-making processes through personality traits. In line with the results obtained, the second research question, which personality traits and decision-making styles are related, was answered.

When the relationships between personality traits and the CDDQ are analyzed in Table 5, it is found that neurotic personality traits show positive relationships with readiness (*β* = 0.086, *p* = 0.002), a lack of information (*β* = 0.180, *p* < 0.000), and inconsistent information (*β* = 0.137, *p* = 0.005). This indicates that neurotic individuals experience more difficulties in terms of access to information and decision readiness in decision processes. On the other hand, conscientiousness showed negative relationships with inconsistent information (*β* = −0.215, *p* = 0.004) and a lack of information (*β* = −0.252, *p* < 0.000). This suggests that conscientious individuals plan more in their decision-making processes and are more successful in accessing information. Similarly, openness to experience has a negative relationship with a lack of information (*β* = −0.111, *p* = 0.012), suggesting that open individuals have less difficulty in accessing information. Lastly, agreeableness showed negative relationships with inconsistent information (*β* = −0.122, *p* = 0.004). This may indicate that agreeable people react less to complex and inconsistent information and remain calmer in the face of inconsistent information. In line with the results obtained, the first research question, which personality traits are associated with career decision-making difficulties, was answered.

When the relationships between GDMSs and the CDDQ were analyzed in Table 5, it was concluded that the rational decision-making style showed a positive relationship with a lack of readiness (*β* = 0.081, *p* = 0.006) but a negative relationship with inconsistent information (*β* = −0.114, *p* = 0.002). This suggests that rational individuals are successful in evaluating information but may experience a lack of readiness. The dependent decision-making style showed positive relationships with a lack of readiness (*β* = 0.122, *p* < 0.000), a lack of information (*β* = 0.145, *p* = 0.002), and inconsistent information (*β* = 0.087, *p* = 0.043), indicating that dependent individuals need more support in their decision processes. The avoidant decision-making style shows strong positive relationships with a lack of readiness (*β* = 0.186, *p* < 0.000), a lack of information (*β* = 0.200, *p* < 0.000), and inconsistent information (*β* = 0.179, *p* < 0.000). This suggests that avoidant individuals tend to avoid decision processes. The spontaneous decision-making style is also positively related to a lack of readiness (*β* = 0.087, *p* = 0.001), a lack of information (*β* = 0.157, *p* = 0.002), and inconsistent information (*β* = 0.116, *p* = 0.012), indicating that spontaneous individuals are more likely to experience a lack of information and readiness. In summary, the rational decision-making style is positively associated with a lack of readiness and negatively associated with inconsistent information. The dependent decision-making style is positively associated with all career decision-making difficulties. The avoidant decision-making style is positively associated with all career decision-making difficulties. The coefficients of the significant relationships between the avoidant decision-making style and the dimensions of career difficulties were found to be higher than those of the other decision-making styles. This indicates that individuals with an avoidant decision-making style experience more career decision-making difficulties. The spontaneous decision-making style is positively correlated with all career decision-making difficulties.

When the path analysis results were evaluated, in general, it was seen that personality traits explained 19% of the variance associated with rational decision-making, 12% of the variance associated with spontaneous decision-making, 0.9% of the variance associated with spontaneous decision-making, 0.7% of the variance associated with avoidant decision-making, and 0.6% of the variance associated with dependent decision-making. In addition, personality traits and decision-making styles explained 22% of the variance associated with inconsistent information, personality traits and decision-making styles explained 23% of the variance associated with a lack of information, and personality traits and decision-making styles explained 28% of the variance associated with a lack of readiness. It can be said that the explained variance in career decision-making difficulty is obtained by the direct and indirect effects of personality traits and the direct effect of decision-making styles.

When personality traits and CDDQ dimensions are analyzed, it is seen that the most influenced CDDQ dimensions are the lack of information and inconsistent information. A lack of information and inconsistent information are influenced more by conscientiousness. When the GDMSs and CDDQ dimensions are analyzed, it is seen that a lack of information, a lack of readiness, and inconsistent information are influenced more by the avoidant style.

### 4.5. The Mediating Role of GDMSs

The values of the indirect effects between the variables in the research model are presented in Table 6. Indirect effects are situations where the effect of one variable on another variable is realized through one or more mediating variables. This analysis, as an extension of path analysis, examines how the effects of independent variables on dependent variables are shaped through mediating relationships. The identification of indirect effects provides an important perspective independent of the main effects by revealing the more complex relationships of the model.

GDMSs played a significant role in the relationship between the neuroticism and openness to experience personality traits and the CDDQ. Similarly, GDMSs played a significant role in the relationship between the conscientiousness personality trait and the lack of information and inconsistent information dimensions. On the other hand, while the direct relationship between the extraversion and agreeableness personality traits and the CDDQ was significant, there was no significant relationship through GDMSs. These findings suggest that GDMSs play a mediating role in the relationship between personality traits and the CDDQ. According to the results of the analysis, some personality traits have direct effects on decision-making processes, while others may have indirect effects through GDMSs. In line with the results obtained, the third research question, what role do decision-making styles play in the relationship between personality traits and career decision-making difficulties, was answered.

## 5. Discussion

According to the results of the research model, statistically significant relationships were observed between personality traits and decision-making styles. Extraverted individuals were found to be negatively related to rational and avoidant decision-making and positively related to spontaneous decision-making. On the other hand, agreeableness was found to be positively associated with rational and dependent decision-making and negatively associated with avoidant and spontaneous decision-making. The conscientiousness personality trait was positively associated with rational and intuitive decision-making and negatively associated with dependent and avoidant decision-making. The neurotic personality type was positively associated with dependent and intuitive decision-making. Finally, the openness to experience personality type was positively associated with rational and intuitive decision-making and negatively associated with avoidant decision-making. These results are similar to the findings of previous studies (Riaz et al., 2012; Narooi & Karazee, 2015; El Othman et al., 2020).

### 5.1. The Relationship Between CDDQ and Personality

Studies have shown that personality traits are variables that significantly explain career indecision (Di Fabio et al., 2012, 2015; Martincin & Stead, 2015; Akbar et al., 2023). In our study, no significant relationship was found between extraversion and career decision-making difficulties. However, Di Fabio et al. (2012) and Di Fabio and Saklofske (2014) found that the extraversion personality trait negatively and significantly affected three of the career decision-making difficulties (lack of readiness, lack of information, inconsistent information). Other studies have also revealed that extraverted individuals experience less career indecision (Di Fabio et al., 2015; Martincin & Stead, 2015; Akbar et al., 2023). In addition, studies conducted by Lounsbury et al. (2005), Salter (2008), Feldt and Woelfel (2009), Hirschi (2009), Gunkel et al. (2010), Feldt et al. (2010), and Smith (2011) found no significant relationship between extraversion and career decision-making difficulties.

In our study, the neurotic personality trait was found to be statistically significantly and positively associated with a lack of information. Di Fabio et al. (2012) found that the neurotic personality trait was positively and significantly related to the lack of readiness and inconsistent information dimensions. Similarly, many studies have shown that neuroticism is positively related to career indecision (Burns et al., 2013; Martincin & Stead, 2015; Di Fabio et al., 2015; Di Fabio & Saklofske, 2014; Öztemel, 2014; Akbar et al., 2023). Our study reveals consistent results with these studies in the literature.

In our study, it was concluded that conscientiousness and career decision-making difficulties (lack of readiness, lack of information, inconsistent information) were negatively and significantly associated. These results are consistent with the studies in the literature (Di Fabio & Palazzeschi, 2012; Öztemel, 2014; Martincin & Stead, 2015).

Finally, openness to experience, one of the personality traits, was found to be significantly and negatively associated with the lack of information dimension. Similarly, Di Fabio et al. (2015) found a negative and significant relationship between openness to experience and a lack of information. However, Di Fabio et al. (2012) did not find a significant relationship between openness to experience and a lack of information. In addition, no significant relationship was found between openness to experience and the entire career decision-making difficulties scale in the studies conducted by Albion and Fogarty (2002), Salter (2008), Feldt and Woelfel (2009), Feldt et al. (2010), and Gati et al. (2011).

In general, the findings show that the neurotic personality trait increases career decision-making difficulties, whereas the conscientiousness and openness to experience traits decrease career decision-making difficulties.

### 5.2. The Relationship Between CDDQ and GDMSs

In the present study, all decision-making styles except the intuitive decision-making style were statistically significantly associated with at least one dimension of career decision-making difficulties. In the literature, limited studies were found in which decision-making styles and career decision-making difficulties were investigated together. In the study conducted by Chong and Tan (2019), significant and positive relationships were found between all decision-making styles except the rational decision-making style and career decision-making difficulties in which all dimensions were considered together. The strongest of these relationships was found between the avoidant decision-making style and career decision-making difficulties (*r* = 0.425; *p* < 0.000). This finding is consistent with our study.

### 5.3. Limitations of This Study

This study has some limitations. First, the fact that the data were collected only from university students makes it difficult to generalize the results to different age groups, occupational groups, or educational levels. This situation narrows the scope of the findings of this study. In addition, the fact that the sample was limited to a single cultural context limits the evaluation of how the effects of personality traits and decision-making styles may vary in different cultural contexts. The cross-sectional design of this study makes it difficult to clearly identify cause-and-effect relationships, while at the same time, it eliminates the possibility of monitoring the changes in these relationships over time. In addition, the fact that the data are based on participants’ self-reports may increase the risk of social desirability bias or misrepresentation, which can be considered as another important limitation that may affect the accuracy of the findings.

In order to overcome these limitations in future studies and to contribute to obtaining more comprehensive and generalizable results, a research design covering different age groups, professions, and education levels with a large sample group can be adopted. In addition, conducting the study in more than one cultural context will allow a better understanding of the cultural effects of personality traits and decision-making styles. By using a longitudinal design instead of a cross-sectional design, changes over time and cause–effect relationships can be revealed more clearly.

## 6. Conclusions

In this study, the results show that personality traits and decision-making styles have significant effects on career decision-making difficulties. According to the results of decision-making styles, it was found that the dependent, avoidant, and spontaneous decision-making styles were positively related to a lack of readiness, a lack of information, and inconsistent information, while the rational decision-making style was negatively related to inconsistent information. This result indicates that the rational decision-making style may be more critical in career decision-making. In terms of personality traits, conscientiousness was found to be more influential on career decision-making difficulties than the others. Positive personality traits such as conscientiousness and openness to experience are associated with better coping mechanisms for these difficulties, whereas the neuroticism and avoidant personality traits are linked to greater difficulties. These findings clearly reveal that strategies appropriate to personality traits should be developed in career counseling processes. In line with the data collected from university students, it is thought that comprehensive program recommendations should be developed for policy-makers to support the career decision-making processes of this group and to minimize the difficulties they face.

Psychological support programs can be integrated into career guidance services for students with neuroticism. These programs should aim to reduce the students’ perception of a lack of information and inconsistent information. Training that focuses specifically on stress management and emotional resilience can help these students overcome their lack of readiness in decision-making processes. For students who tend to make avoidant and spontaneous decisions, it may be useful to provide guidance mechanisms that structure decision-making processes and facilitate access to information.

Developing individualized approaches to career planning among students is important. Workshops that develop leadership skills and encourage analytical thinking can be organized for students who are responsible and open to experience. On the other hand, individual and group-oriented mentoring programs can be implemented to increase the independent thinking skills of students who tend to make dependent decisions. These individualized approaches can help students build on their strengths and overcome their weaknesses.

It is recommended that universities create digital information platforms to reduce the difficulties students face in accessing information. These platforms should provide reliable and up-to-date information on career options, internship opportunities, labor market trends, and post-graduation opportunities. Furthermore, digital tools can be developed to provide step-by-step guidance to students who lack information in their decision-making process. Such tools can help students make more informed career decisions by reducing their uncertainty.

The university curriculum could include courses that develop career planning and decision-making skills for all students. These courses can include case studies and simulation studies for students who tend to make rational decisions. For students with avoidant and dependent decision-making tendencies, group work, self-confidence-building activities, and training that develop leadership skills can be implemented. In this way, students can improve their decision-making skills both individually and through teamwork. Emotional intelligence and stress management training can be an important support mechanism, especially for students with neurotic characteristics. Providing such training at universities will enable students to make career decisions more conscientiously and confidently. For students who tend to make spontaneous decisions, programs can be organized to increase awareness in decision processes.

International exchange programs, internships, and hands-on projects can be encouraged for students who are open to experience. Such programs will support both the personal and professional development of students and increase their capacity for innovative thinking. Creative projects and research can also be encouraged for these students to maximize the benefit of their experience.

Career counseling services should be expanded in universities, and support should be provided in line with students’ personality traits. State-funded career guidance centers should complement these counseling services and aim to solve the problems students face in decision-making processes. Furthermore, evaluation systems with feedback mechanisms can be established in universities to understand and monitor students’ career challenges. These systems can contribute to students’ individual development by considering their personality traits, decision-making styles, and challenges.

In order to make students’ career decision processes easier and more pleasant, they can be supported to achieve rational decision-making instead of dependent, avoidant, or spontaneous decision-making. In terms of personality traits, conscientiousness, agreeableness, openness to experience, and related skills can be supported. Finally, although this study focuses specifically on students who experience difficulties in the decision-making process, it is important to consider that career decisions present different challenges and opportunities for each student. These recommendations will facilitate university students in making more informed career decisions and support them in participating in the labor market in a more prepared manner after graduation. Thus, more efficient and effective results can be achieved at both individual and societal levels.

## Figures and Tables

**Table 1:** Relationship between personality traits and GDMSs.

	Rational	Intuitive	Dependent	Avoidant	Spontaneous
Extraversion	+/−	+	−	−	+
Conscientiousness	+	+/−	+/−	−	−
Agreeableness	+	+/−	+/−	−	−
Neuroticism	−	+	+	+/−	+/−
Openness	+	+	−	−	ns

Note: (+) positive; (−) negative; (ns) non-significant.

**Table 2:** Means, standard deviations, and Cronbach’s alpha coefficients.

Scales	Items	Means	Standard. Dev.	Cronbach’s Alpha
**Personality traits**				
Extraversion	8	26.56	5.83	0.82
Conscientiousness	9	33.02	5.67	0.77
Agreeableness	9	32.39	5.21	0.66
Neuroticism	8	25.19	6.11	0.79
Openness to experience	10	35.69	6.14	0.78
**GDMSs**				
Rational	5	20.29	3.66	0.84
Intuitive	5	18.43	4.08	0.83
Dependent	4	13.76	3.67	0.82
Avoidant	5	12.37	5.20	0.89
Spontaneous	5	13.04	4.67	0.80
**CDDQ**				
Lack of readiness	12	35.44	6.19	0.57
Lack of information	12	31.34	12.59	0.95
Inconsistent information	10	24.78	9.45	0.91

**Table 3:** Correlations.

	1	2	3	4	5	6	7	8	9	10	11	12
1—Extraversion	1											
2—Conscientiousness	0.282 **	1										
3—Agreeableness	0.136 **	0.243 **	1									
4—Neuroticism	−0.255 **	−0.293 **	0.020	1								
5—Openness	0.390 **	0.252 **	0.144 **	−0.189 **	1							
6—Rational	0.088 *	0.393 **	0.189 **	−0.090 *	0.240 **	1						
7—Intuitive	0.141 **	0.110 *	0.089 *	0.095 *	0.251 **	0.212 **	1					
8—Dependent	−0.097 *	−0.117 **	0.152 **	0.234 **	−0.008	0.158 **	0.085	1				
9—Avoidant	−0.211 **	−0.478 **	−0.129 **	0.365 **	−0.205 **	−0.287 **	−0.011	0.262 **	1			
10—Spontaneous	0.082	−0.290 **	0.169 **	0.119 **	0.032	−0.314 **	0.103 *	0.010	0.412 **	1		
11—Lack of readiness	−0.165 **	−0.271 **	−0.013	0.297 **	−0.056	−0.025	0.079	0.334 **	0.468 **	0.265 **	1	
12—Lack of information	−0.178 **	−0.371 **	−0.080	0.306 **	−0.159 **	−0.195 **	−0.006	0.228 **	0.420 **	0.274 **	0.484	1
13—Inconsistent information	−0.147 **	−0.380 **	−0.161 **	0.263 **	−0.119 **	−0.269 **	0.017	0.154 **	0.408 **	0.286 **	0.452	0.820

* *p* < 0.05; ** *p* < 0.01.

**Table 4:** Demographic characteristics (N = 505).

	Frequency	%
**Gender**		
Female	319	63.2
Male	186	36.8
**Grade**		
First	115	22.8
Second	112	22.2
Third	125	24.8
Fourth	153	30.3
**Difficulty in career decision-making**		
Yes	315	62.4
No	190	37.6
**Intentionally choosing the field of study**		
Yes	369	73.1
No	136	26.9

**Table 5:** Estimated model results.

Path	*β*	*p*	Result
**Personality traits and GDMSs**			
Extraversion → rational	−0.091	0.034	Accepted
Extraversion → avoidant	−0.174	0.007	Accepted
Extraversion → spontaneous	0.225	<0.000	Accepted
Agreeableness → rational	0.109	0.037	Accepted
Agreeableness → dependent	0.278	<0.000	Accepted
Agreeableness → avoidant	−0.160	0.041	Accepted
Agreeableness → spontaneous	−0.186	0.005	Accepted
Conscientiousness → rational	0.409	<0.000	Accepted
Conscientiousness → dependent	−0.166	0.011	Accepted
Conscientiousness → spontaneous	−0.428	<0.000	Accepted
Conscientiousness → intuitive	0.121	0.037	Accepted
Neuroticism → dependent	0.147	0.003	Accepted
Neuroticism → intuitive	0.180	<0.000	Accepted
Openness to experience → rational	0.217	<0.000	Accepted
Openness to experience → avoidant	−0.280	<0.000	Accepted
Openness to experience → intuitive	0.345	<0.000	Accepted
**Personality traits and CDDQ**			
Neuroticism → lack of readiness	0.086	0.002	Accepted
Neuroticism → lack of information	0.180	<0.000	Accepted
Neuroticism → inconsistent information	0.137	0.005	Accepted
Agreeableness → inconsistent information	−0.122	0.004	Accepted
Conscientiousness → lack of information	−0.252	<0.000	Accepted
Conscientiousness → inconsistent information	−0.215	0.001	Accepted
Openness to experience → lack of information	−0.111	0.012	Accepted
**GDMSs and CDDQ**			
Rational → lack of readiness	0.081	0.006	Accepted
Rational → inconsistent information	−0.114	0.002	Accepted
Dependent → lack of readiness	0.122	<0.000	Accepted
Dependent → lack of information	0.145	0.002	Accepted
Dependent → inconsistent information	0.087	0.043	Accepted
Avoidant → lack of readiness	0.186	<0.000	Accepted
Avoidant → lack of information	0.200	<0.000	Accepted
Avoidant → inconsistent information	0.179	<0.000	Accepted
Spontaneous → lack of readiness	0.087	0.001	Accepted
Spontaneous → lack of information	0.157	0.002	Accepted
Spontaneous → inconsistent information	0.116	0.012	Accepted

Note: significant relationships are displayed.

**Table 6:** Indirect effects of personality types on CDDQ.

Path	Indirect Effect	CI	*p*
Extraversion → lack of readiness	−0.027	[−0.075; 0.023]	0.311
Extraversion → lack of information	0.000	[−0.039; 0.038]	0.955
Extraversion → inconsistent information	0.004	[−0.038; 0.040]	0.853
Agreeableness → lack of readiness	−0.003	[−0.059; 0.046]	0.841
Agreeableness → lack of information	−0.012	[−0.047; 0.026]	0.610
Agreeableness → inconsistent information	−0.024	[−0.061; 0.012]	0.241
Conscientiousness → lack of readiness	−0.027	[−0.078; 0.010]	0.154
Conscientiousness → lack of information	−0.055	[−0.092; −0.022]	0.002
Conscientiousness → inconsistent information	−0.074	[−0.116; −0.039]	0.002
Neuroticism → lack of readiness	0.025	[0.008; 0.048]	0.002
Neuroticism → lack of information	0.016	[0.004; 0.039]	0.002
Neuroticism → inconsistent information	0.011	[0.001; 0.035]	0.020
Openness to experience → lack of readiness	−0.039	[−0.080; −0.002]	0.042
Openness to experience → lack of information	−0.033	[−0.081; −0.014]	0.001
Openness to experience → inconsistent information	−0.050	[−0.039; −0.025]	0.002

## Data Availability

The raw data supporting the conclusions of this article will be made available by the authors upon request.
